# Sense and Learn: Recent Advances in Wearable Sensing and Machine Learning for Blood Glucose Monitoring and Trend-Detection

**DOI:** 10.3389/fbioe.2022.876672

**Published:** 2022-05-12

**Authors:** Ahmad Yaser Alhaddad, Hussein Aly, Hoda Gad, Abdulaziz Al-Ali, Kishor Kumar Sadasivuni, John-John Cabibihan, Rayaz A. Malik

**Affiliations:** ^1^ Department of Mechanical and Industrial Engineering, Qatar University, Doha, Qatar; ^2^ KINDI Center for Computing Research, Qatar University, Doha, Qatar; ^3^ Weill Cornell Medicine - Qatar, Doha, Qatar; ^4^ Center for Advanced Materials, Qatar University, Doha, Qatar

**Keywords:** diabetes mellitus, non-invasive wearables and sensors, hypoglycemia, machine learning, blood glucose management, deep learning, bodily fluids glucose

## Abstract

Diabetes mellitus is characterized by elevated blood glucose levels, however patients with diabetes may also develop hypoglycemia due to treatment. There is an increasing demand for non-invasive blood glucose monitoring and trends detection amongst people with diabetes and healthy individuals, especially athletes. Wearable devices and non-invasive sensors for blood glucose monitoring have witnessed considerable advances. This review is an update on recent contributions utilizing novel sensing technologies over the past five years which include electrocardiogram, electromagnetic, bioimpedance, photoplethysmography, and acceleration measures as well as bodily fluid glucose sensors to monitor glucose and trend detection. We also review methods that use machine learning algorithms to predict blood glucose trends, especially for high risk events such as hypoglycemia. Convolutional and recurrent neural networks, support vector machines, and decision trees are examples of such machine learning algorithms. Finally, we address the key limitations and challenges of these studies and provide recommendations for future work.

## Graphical Abstract

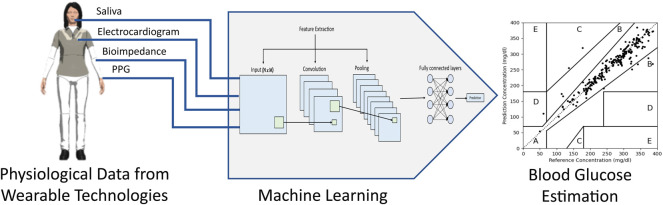

## 1 Introduction

Blood glucose levels are closely regulated within a desirable range by several hormones released primarily by the pancreas. Diabetes mellitus is a group of metabolic diseases characterized by elevated glucose levels (American Diabetes Association, 2013). The 2021 International Diabetes Federation (IDF) atlas has estimated that there are 537 million adults with diabetes and that number is expected to increase to 784 million by 2045. Type 1 diabetes affects between 5 and 10% of patients and is characterized by a lack of insulin production and a higher variability in blood sugars, which requires exogenous insulin and more regular monitoring of blood glucose (American Diabetes Association, 2020). Type 2 diabetes affects up to 90% of all patients diagnosed with diabetes and can be further subdivided into a distinct number of subtypes with varying degrees of insulin deficiency, insulin resistance, and propensity for developing complications (Ahlqvist et al., 2020; Ahlqvist et al., 2018).

Normal blood glucose concentrations should lie within a range of 4.0–5.5 mmol/L (72–99 mg/dl) after an 8 h fast and should be < 7.8 mmol/L (140 mg/dl) 2 h after eating (Güemes et al., 2015). Chronic hyperglycemia is associated with long-term microvascular and macrovascular complications (de Boer, 2008). Hypoglycemia occurs due to excess insulin which can be exogenous or endogenous leading to glucose dropping below the normal range. Hypoglycemia occurs in both type 1 diabetes and type 2 diabetes (Briscoe, 2006; Zammitt and Frier, 2005; Miller et al., 2001). There are clear American Diabetes Association (ADA) clinical practice recommendations to achieve optimal blood glucose targets using different therapies (American Diabetes Association, 2020a; American Diabetes Association, 2020b), but are inherently associated with an increased risk of hypoglycemia (Ohkuma et al., 2021), especially in patients who have reduced awareness of hypoglycemia (Yeoh et al., 2015).

The ADA categorizes hypoglycemia into: Level 1 with a blood-glucose value between 54 mg/dl and 70 mg/dl; level 2 with a blood-glucose value less than 54 mg/dl; and level 3 with severe hypoglycemia characterized by altered mental and/or physical status requiring external assistance (American Diabetes Association et al, 2021). The symptoms of hypoglycemia are categorized as autonomic, with adrenergic (tremor, palpitations, tachycardia, and anxiety) and cholinergic (sweating, hunger, and paresthesia) manifestations. Neuroglycopenic symptoms may include dizziness, weakness, drowsiness, delirium, confusion, seizure, and coma. The blood-glucose threshold at which an individual patient will experience hypoglycemic symptoms depends on overall glycemic control as the threshold for hypoglycemia will be higher in patients with consistently elevated blood-glucose. Thus, self-monitoring of blood glucose is a key part of diabetes management (Diouri et al., 2021).

The most widely used glucose monitoring systems rely on disposable test strips that read the blood glucose from a sample obtained using the finger prick approach. This method is associated with discomfort, especially when it is undertaken frequently in patients with type 1 diabetes (Heinemann, 2008). Continuous glucose monitoring (CGM) devices measure interstitial glucose and have been shown to marginally improve overall glycemic control and reduce the incidence of hypoglycemia (Nathanson et al., 2021; Lee I. et al., 2021), but suffer from several inherent limitations. There is a time lag between the interstitial glucose measured by CGM and actual blood glucose and there are inconsistencies in the readings of different CGM devices (Kamath et al., 2009; Damiano et al., 2012). Furthermore, most CGMs are relatively expensive, which limits the wider adoption of such technology among diabetic patients.

**FIGURE 1:**
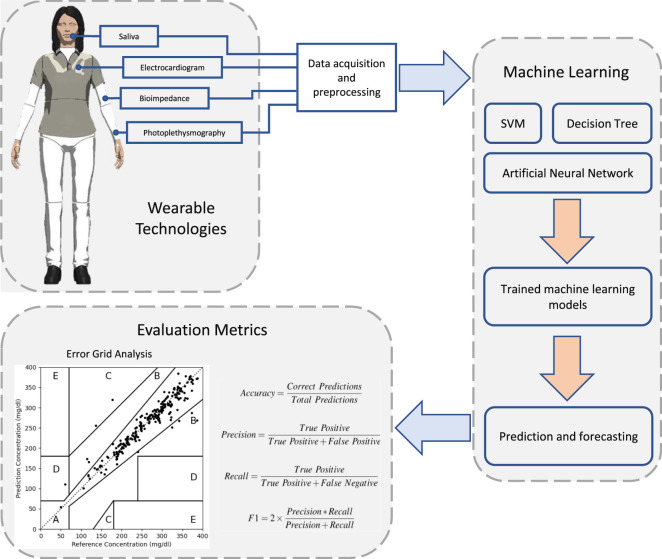
An overview of glucose monitoring systems that are based on wearable sensing technologies and machine learning techniques. Wearable devices collect different physiological signs using different sensor technologies. Machine learning algorithms are then used to train and develop prediction models. The predictive performance of these models is assessed using evaluation metrics against actual recorded blood glucose levels.

Wearables are lightweight devices capable of measuring different vital signs and modalities such as heart rate, temperature, respiration rate, activity level, and skin conductance (Cheol Jeong et al., 2019). Wearable technologies have been proposed to improve the quality of life by collecting and sharing data between people and their carers (Seneviratne et al., 2017). Wearable devices have different functions and may include patches, belts, lenses, earphones, socks, glasses, watches, wrist bands, and bracelets (Dunn et al., 2018). Wearables have been recommended in the elderly to increase their independence and have been proposed for autism screening and therapy (Kekade et al., 2018; Cabibihan et al., 2016; Cabibihan et al., 2018). Recently, the readings from wearable technologies have been associated with blood glucose levels (Bekkink et al., 2019; Corino et al., 2017; Maritsch et al., 2020). Using such devices to indirectly monitor glucose represents a non-invasive, convenient, and inexpensive method over CGM. A typical glucose monitoring system consists of wearable devices and machine learning models assessed against FDA established evaluation metrics (Figure 1).

Siddiqui et al. surveyed different types of non-invasive blood glucose monitoring equipment and highlighted the potential role of artificial intelligence in solving some of the existing challenges (Siddiqui et al., 2018). Non-invasive monitoring of glucose levels from breath samples and epidermal electrochemical glucose sensors hold promise (Lekha and Suchetha, 2020; Kim et al., 2018). Other non-invasive sensors that measure physiological parameters such as pulse and pulse pressure may help to improve glucose management among diabetic patients, especially during physical activity (Ding and Schumacher, 2016). A wide range of non-invasive techniques for blood glucose monitoring have been developed utilizing glucose monitoring in bodily fluids (Nawaz et al., 2016; Bruen et al., 2017).

Despite the existence of many studies related to non-invasive glucose monitoring, several questions remain unanswered:

1. What technologies can be used in blood glucose prediction and trends detection?
2. Which machine learning algorithms can be used?
3. To what extent are these technologies and techniques successful?
4. What are the limitations of the existing approaches?

To answer these questions, we have reviewed the advances over the past five years in non-invasive sensors, wearable technologies, and machine learning approaches to estimate blood glucose, especially to identify hypoglycemia. We have identified the major limitations and challenges faced by these studies and provided recommendations to overcome these constraints. We have reviewed studies that have developed non-invasive sensors and wearable devices as well as contributions that have employed existing commercially available devices. Studies without working prototypes were excluded. The considered search keywords included *non-invasive* or *noninvasive*, *sensor*, *wearable*, *glucose*, *levels*, *trends*, *hypoglycemia*, *monitoring*, *machine learning*, and *prediction*.

## 2 Non-invasive Sensors and Wearables

**FIGURE 2:**
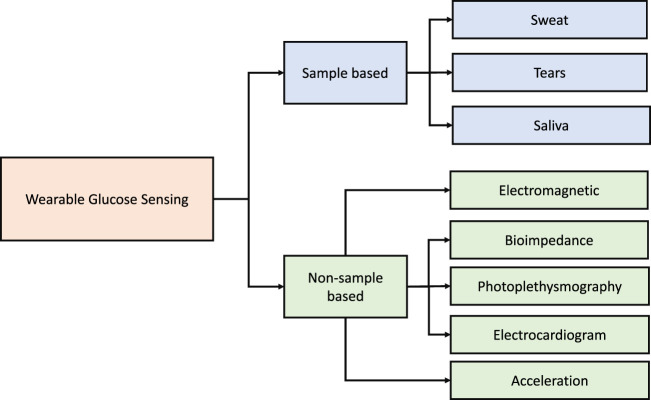
A broad classification of the wearable and sensor technologies considered in glucose monitoring over the past five years.

**FIGURE 3:**
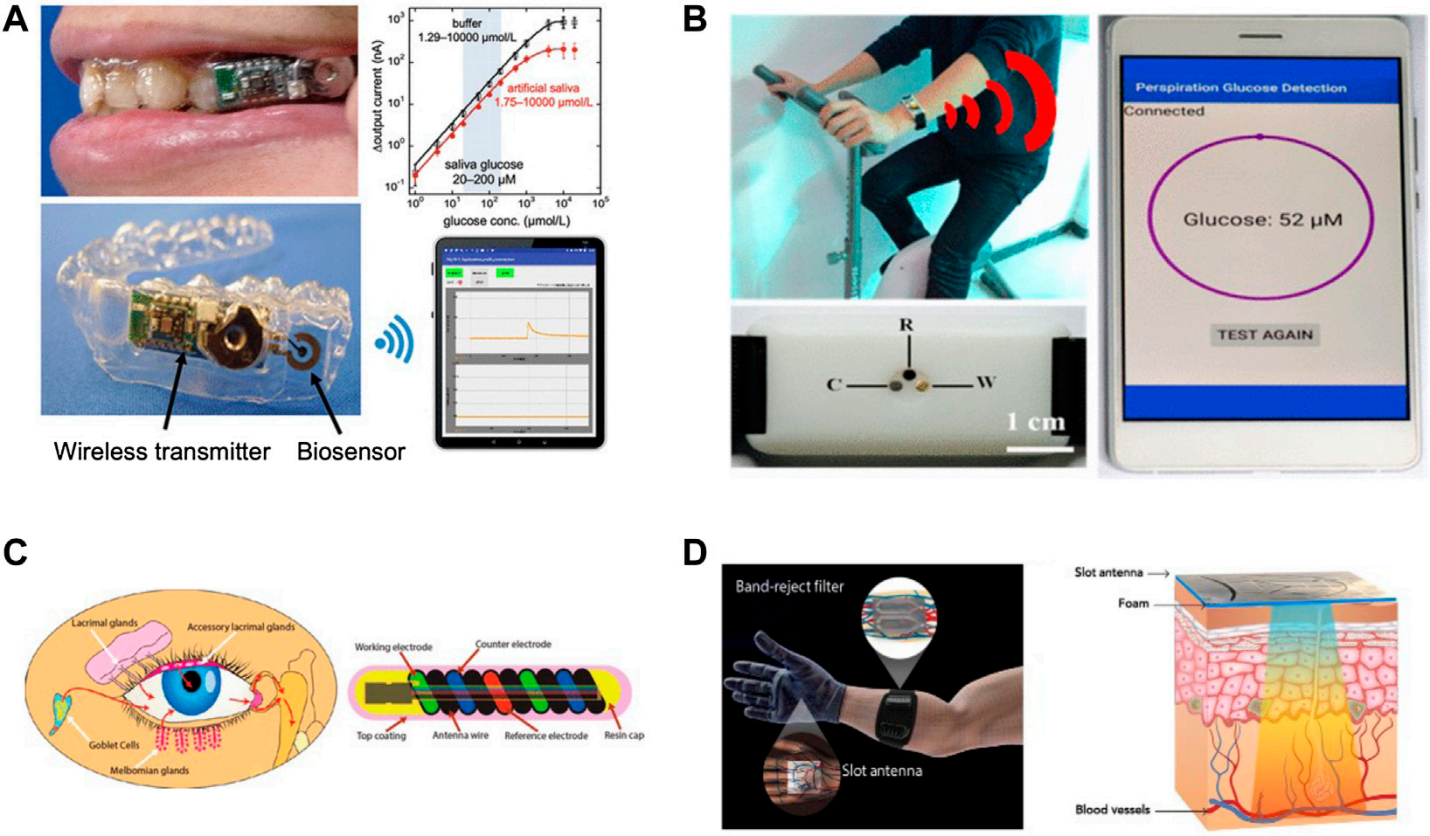
Examples of developed wearable devices and sensors that are aimed to aid in blood glucose management. **(A)** A wearable mouthguard biosensor that measures salivary glucose levels (Adapted with permission from Arakawa et al. (2020)). **(B)** Wearable sensor to analyze perspiration glucose (Adapted with permission from Zhu X. et al. (2018). **(C)** A biosensor that can measure tear glucose levels (Adapted with permission from Kownacka et al. (2018)). **(D)** An electromagnetic wearable glove for continuous glucose monitoring (Adapted with permission from Hanna et al. (2020)).

This section presents the advances in wearables and sensors over the past five years. The technologies used have varied from direct measurement of glucose from bodily fluids such as saliva, sweat, and tears to the detection of physiological variables which change with blood glucose (Figure 2). Examples of such wearable devices and sensors are illustrated in Figure 3. A summary of the surveyed studies is provided in Table 1.

**TABLE 1:** A summary of the advances in non-invasive sensors and wearable technologies for blood glucose monitoring.

Study	Device	Experiments	Key Findings	Limitations
Susana et al. (2022)	PPG device	Collected data from 80 participants	The classification of blood glucose levels into normal and diabetic	Limited experimental settings and no reported instances of hypoglycemia
Chu et al. (2021)	PPG device	PPG and physiological data from 2,538 participants	Promising blood glucose prediction performance for the group without medication	Limited PPG data collected from each participant with limited results for the group with medication
Maritsch et al. (2020)	PPG wrist wearable	The data was obtained from one participant with T1DM	The detection of hypoglycemia using HRV time features	Limited to one participant
Porumb et al. (2020b)	ECG chest wearable device	The study acquired the ECG data from healthy participants monitored for up to 14 days	The ability to detect nocturnal hypoglycemia relying on raw ECG signal	No participants with diabetes and limited instances of hypoglycemia
Bekkink et al. (2019)	ECG patch wearable device	The data was acquired from patients with T1DM	The identification of HRV patterns in relation to early detection of hypoglycemia	Limited instances of hypoglycemia
Charamba et al. (2021)	12-lead ECG	ECG, CGM, and diary from 17 patients with type 1 diabetes	Identifying a negative relationship between QTC and hypoglycemia	No female participants and no investigation on the effects of medications
Li et al. (2021)	ECG device	ECG and glucose data from 21 participants	An overall accuracy of 81.69% in classifying blood glucose to three groups	Limited to young participants and small sample size
Saha et al. (2017)	EM patch antennas	Tested with water-based samples and with blood samples from humans	The detection of glucose spikes in humans	Limited to experimental settings and susceptible to noise
Hanna et al. (2020)	EM wearable glove	Glucose solutions and healthy participants	High correlation with glucose changes and blood glucose trends	No diabetic patients and limited testing conditions
Raj et al. (2021)	EM antenna	Tested with plasma glucose	Deviations in the reflection coefficient	Limited experiments with participants
Zidane et al. (2021)	EM ring	Tested with glucose solutions	The ability to detect glucose with a 1.665 mmol/L resolution	No experiments with participants were reported
Zapasnoy et al. (2021)	Microwave sensor	Tested with glucose solutions	Detecting glucose concentrations in sodium chloride solutions with a 1 mmol/L resolution	No testing with participants were reported
Liu et al. (2016)	Bioimpedance antenna	*In vitro* test in a pork slab	Glucose levels changes can be picked up by bioimpedance parameters	Limited testing conditions with a prototype
Nanayakkara et al. (2018)	Bioimpedance and near-infrared	Datasets from one subject	High correlation was achieved	Testing was limited to one participant and no blood trends
Tronstad et al. (2019)	Bioimpedance	Data were collected from 20 patients with T1DM	Bioimpedance aids in the prediction	Limited experimental settings and no blood trends
Snekhalatha et al. (2018)	Bioimpedance to measure galvanic skin response	Data were collected from 50 participants with diabetes and 50 healthy participants	Negative correlation was observed with blood glucose levels	Limited to experimental settings and no blood trends
Sanai et al. (2021)	Bioimpedance wearable ring	Tested with 14 patients with type 2 diabetes	Reported accurate prediction of blood glucose levels	No testing with patients with type 1 diabetes
Xiao et al. (2019)	Sweat glucose wearable sensor	The device was tested with 4 participants	Dynamic detection range	Testing only with healthy participants and no blood trends
Xuan et al. (2018)	Sweat glucose biosensor	Tested with sweat samples	High linearity and short response time	Limited experimental testing with a prototype
Zhu et al. (2018b)	Wristband sweat glucose wearable	Tested using sweat samples and with volunteers	Continuous sweat glucose monitoring with a mobile app	Limited testing conditions, no patients with diabetes, and no blood trends
Zhao et al. (2019)	Sweat glucose smartwatch	Tested using sweat samples and with 6 healthy participants	Real-time glucose monitoring using a smartwatch	No participants with diabetes and no blood trends
Katseli et al. (2021)	Sweat wearable ring	Tested with one healthy participant	Detecting sweat glucose in the range of 12.5–400 *μ*mol/L	Limited testing conditions
Sempionatto et al. (2021)	Touch-based sweat sensor	Tested with three healthy participants	High correlation with blood glucose	Not tested with patients with diabetes
Kim et al. (2017)	Contact lens tears glucose sensor	Tested in rabbit and bovine eyes	Wireless detection of tears glucose	No reported tests with participants
Park et al. (2018)	Soft and smart tears glucose lens	Tested on a live rabbit	Real-time detection of tears glucose wirelessly	No reported tests with participants
Elsherif et al. (2018)	Optical tears glucose sensor	Tested with an artificial eye and different glucose concentrations	Detection of glucose concentration using smartphone camera	No *in vivo* tests or with participants
Kownacka et al. (2018)	Flexible tears glucose biosensor	Tested with 6 participants	Achieved decent performance on Clarke’s error grid	No blood trends and no diabetic participants
Lee et al. (2021b)	Tears glucose contact lens	Tested using glucose solutions and human tears from three participants	Detecting tear glucose with a detection limit of 211 nM	No tests with diabetic patients
Geelhoed-Duijvestijn et al. (2021)	Tears glucose biosensor	Evaluated with 24 patients with type 1 diabetes	Comparable performance to that of CGM	Requires further evaluations with blood glucose trends
Lin et al. (2017)	Saliva glucose sensor	Tested with saliva samples from 9 participants	Saliva glucose detection range that corresponded to blood glucose changes	Limited testing conditions and no tests with diabetic patients
Chen et al. (2019)	Saliva glucose smart toothbrush	Saliva samples collected from 5 subjects	Linear detection range and reflected with blood glucose changes	Limited to healthy participants
Arakawa et al. (2020)	Saliva glucose mouthguard	Tested with artificial saliva	Detected glucose concentration	Limited testing conditions and no *in vivo* tests
Zhang et al. (2021)	Salivary glucose biosensor	Evaluated with saliva samples	Good linearity for glucose concentration between 0 and 50 mg/L	Further evaluations are required with patients with diabetes
Abbas et al. (2018)	Fingertip wearable with an accelerometer	Simulated hand tremors	Detection of tremors that are similar to that exhibited during hypoglycemia	Limited tests and no patients with diabetes
Aljihmani et al. (2019)	Wrist wearable with an accelerometer	Data collected from seven subjects with T1DM and T2DM	Identifying tremors under fatigue	No hypoglycemic events
Jaggers et al. (2019)	Smartwatch with an accelerometer	Data was acquired from 10 patients with T1DM	Activity recognition helps in the prediction of nocturnal hypoglycemia	Limited to adolescent subjects with T1DM and nocturnal hypoglycemia

### 2.1 Photoplethysmography

Heart rate variability (HRV) reflects physiological changes in the human body and provides insights on cardiac health and the autonomic nervous system, which can be used as an indicator of current or impending diseases (Yuan et al., 2020). For example, it was considered as a predictor in rapid renal function deterioration (Chou et al., 2019), sudden cardiac death (Sessa et al., 2018), and stroke or post-stroke complications (Lees et al., 2018). The analysis of HRV relies on methods in either time-domain or frequency domain. The root mean square of successive differences (RMSSD), the standard deviation of normal to normal interval (SDNN), and average heart rate (HR) are widely used time domain indices (Acharya et al., 2006). High frequency (HF), low frequency (LF) and very low-frequency (VLF) are examples of frequency-domain parameters. HRV has been associated with the severity of hypoglycemia and fluctuation of interstitial glucose (Silva et al., 2016; Klimontov et al., 2016).

Estimating HRV parameters from wearables to predict hypoglycemia has been investigated in previous studies with changes in HRV occurring up to 90 min prior to hypoglycemia (Bekkink et al., 2019). Empatica E4 (Empatica Inc., US) is one example of a wearable used to estimate HRV parameters utilizing a photoplethysmography (PPG) sensor to measure the blood volume pulse. The PPG sensor in Empatica E4 has been shown to provide accurate arrhythmia classification (Corino et al., 2017). A previous study has demonstrated that Empatica E4 can be used to detect the occurrence of hypoglycemia (Maritsch et al., 2020). The study collected HRV time features (e.g., RMSSD) extracted from the recorded inter-beat intervals from the wearable device and interstitial glucose readings using CGM (i.e., FreeStyle Libre) from a participant with T1DM. Using a machine learning model based on decision trees (i.e., gradient boosting), the developed model was able to predict hypoglycemia in the test set with an accuracy of 82.7%. The study had major limitations in terms of the features considered, physiological signals, and was only tested on one participant. However, the initial results demonstrated that wearables can indeed be used to predict hypoglycemic events using HRV parameters.

A recent study demonstrated the application of a PPG device in the classification of blood glucose levels into normal and diabetic (Susana et al., 2022). With PPG signal and blood glucose data collected from 80 participants, the study reported an accuracy of 98% with a decision tree based classifier. Another recent study investigated blood glucose prediction based on PPG and physiological data acquired from 2,538 participants split into two groups with or without medication (Chu et al., 2021). Seventeen features were extracted from the collected data and were used in developing the prediction model. With quarterly measured HbA1c, the best model achieved an accuracy of 94.3% with an RMSE value of 12.4 mg/dl for the group without medication while the results for the group with medication were limited.

### 2.2 Electrocardiogram

Electrical signals arise in the SA node in the right atrium, travel to the atrioventricular node in the interatrial septum and diverge through the left and right bundle of His to the Purkinje fibers terminating in the endocardium and ventricular epicardium causing ventricular contraction. Cardiac electrical activity can be monitored using an Electrocardiogram (ECG). A typical heartbeat is partitioned into three segments, namely PR segment, QRS complex, and ST segment and each segment can be used to identify underlying cardiac conduction defects such as short QT-interval or PR-interval (Nikolaidou et al., 2017; Hasegawa et al., 2016; Kališnik et al., 2019).

The advancement in sensor miniaturization has enabled current wearables to measure changes in cardiac conduction and contraction with good accuracy. The Fitbit Sense smartwatch and Apple watch series 4 are examples of wrist wearables that can measure the heart rate and ECG signal to identify an irregular heart rhythm, e.g., atrial fibrillation. Chest sensors are another trend in wearables that can measure electrical signals from the heart. The Bioharness (3.0, Zephyr Technology, US) is a lightweight, portable chest wearable device that can provide live access to a variety of physiological parameters, including ECG and it has been tested to predict hypoglycemia. The HealthPatch (VitalConnect, San Jose, CA) is another example of an ECG wearable used to derive HRV to investigate hypoglycemia (Bekkink et al., 2019). Other promising wearable ECG devices that provide ease of monitoring include the Bittium Faros (Bittium Corporation, Finland) and Lifetouch (Isansys Lifecare Ltd., United Kingdom).

Changes in blood glucose can alter cardiac repolarization and induce prolongation of the QT interval with an increased risk of cardiac arrhythmia (Kališnik et al., 2019). These changes in cardiac conduction prior to the development of arrythmia could be used to predict the occurrence of hypoglycemia (Ling et al., 2016; Porumb et al., 2020b). In a recent work, the relationship between ECG data and blood glucose in adults has been investigated (Charamba et al., 2021). The study collected the ECG, diary, CGM data for one week from seventeen patients with type 1 diabetes. The study identified a negative relationship between QTC and hypoglycemia.

A recent study utilized an ECG device (i.e., Bioharness) and a glucose monitoring system (i.e., FreeStyle Libre Flash) to develop personalized models to predict the occurrence of nocturnal hypoglycemic events (Porumb et al., 2020b). The study acquired the ECG data from healthy participants monitored for up to 14 days in relation to blood glucose levels below 4 mmol/L to define a hypoglycemia threshold. Using machine learning techniques, the study showed the feasibility of predicting nocturnal hypoglycemia from raw ECG signals. However, the participants in the study were all healthy, hence, low blood glucose instances were limited. Furthermore, the collected data were limited to nocturnal events, thus, ECG circadian changes during the day in relation to glucose concentration were not investigated. Another study acquired ECG and glucose data from 16 healthy adults and 5 with prediabetes to establish a machine learning model based on CNN to classify blood glucose values into three groups, namely, low (i.e., below 6.0 mmol/L), moderate (i.e., above 6.0 mmol/L and below 7.7 mmol/L), and high (i.e., above 7.7 mmol/L) (Li et al., 2021). The results of the best trained model showed an accuracy of 81.69% in classifying the blood glucose values.

### 2.3 Electromagnetic

Non-ionizing parts of the electromagnetic (EM) spectrum (e.g., visible light, radio, and ultraviolet) ranging from 0 to 3.0 PHz have been used for diagnostic and therapeutic medical applications Mattsson and Simkó (2019). Non-invasive estimation of blood glucose has been investigated using electromagnetic waves and near infrared (NIR) waves based on the unique absorption spectrum of glucose (Zhang et al., 2019; Jiang et al., 2017; Hotmartua et al., 2015; Yadav et al., 2014; Tronstad et al., 2019). Transmittance, which measures the scattered light after penetrating the tissue, and reflectance, which measures the reflected light from the skin surface, are two methods that rely on light to acquire information about a substance (Hotmartua et al., 2015). By investigating the properties of both the reflected and transmitted waves, the level of glucose can be estimated (Yadav et al., 2014).

Several devices have been developed that show that changes in EM correlate with glucose concentrations (Cano-Garcia et al., 2016; Shaker et al., 2018). A microstrip patch antenna (1.50 mm × 1.50 mm) utilizing a millimetre band of the EM spectrum has been developed to sense glucose by using two facing antennas operating at 60 GHz to assess the variations in permittivity across the signal path (Saha et al., 2017). The device was capable of detecting changes in small glucose concentrations of 1.33 mmol/L in water-based samples and glucose spikes in humans and therefore has potential applications to detect hypoglycemia. However, the system was limited to experimental settings and was susceptible to noise (e.g., hand motion). Another study demonstrated the design of a compact antenna that operates in the frequency range of 24.1–29.3 GHz and has a bandwidth of 5.2 GHz (Raj et al., 2021). The measured reflection coefficient of the antenna showed deviations due to changes in the electrical properties of plasma glucose, which could potentially be used to measure glucose concentration. Another study demonstrated the design of a circular two cell split ring resonator microwave sensor that displayed sensitivity to changes in glucose concentration in water (Zidane et al., 2021). Their device displayed a glucose detection resolution of 1.665 mmol/L at a frequency of 1.9 GHz. Another study developed a microwave sensor with a relatively wide passband that correlated with blood glucose changes (Zapasnoy et al., 2021). Glucose concentration between 0 and 25 mmol/L in sodium chloride solution could be detected with 1 mmol/L resolution in the frequency range of 1.4–1.7 GHz.

Recently, an innovative EM-based glove wearable device has been developed to monitor blood glucose levels (Hanna et al., 2020). The system consists of two flexible sensors (i.e., slot antenna and a reject filter) and operates in the frequency range of 500 MHz and 3 GHz, which reaches subcutaneous veins and arteries (Costantine et al., 2019). The design of the multiband antenna was made to imitate the vascular anatomy of the hand which improved its sensitivity by concentrating the EM waves on the blood network allowing the monitoring of glucose over a wider frequency range. When the device was tested with glucose solutions at various concentrations, the reflection coefficients varied with the changes in glucose and achieved a high correlation (i.e., greater than 0.90). In *in vivo* experiments, the device showed high correlation (i.e., greater than 0.89) with hypoglycemia and hyperglycemia and reportedly high accuracy on the Clarke’s error grid. Whilst it showed promise in estimating glucose levels in healthy participants, the device is still an experimental prototype that requires extra circuitry and was only tested in controlled conditions without physical activity. Subsequently, the same research team demonstrated high fidelity in serum from 21 participants and achieved 98% accuracy against reference glucose levels (Hanna et al., 2021).

### 2.4 Bioimpedance

Bioimpedance measures the response of a biological medium (e.g., human body) to an electric current. The composition of the biological mediums affects the bioimpedance based on whether they act as insulators, dielectrics, or conductors (Naranjo-Hernández et al., 2019). Hence, bioimpedance measurements can be used to acquire information about body composition, such as fat, muscle, and water and thereby assess obesity (Zhang et al., 2018); sarcopenia in patients with renal disease (Lai et al., 2019), and gastrointestinal disease (Ruiz-Vargas et al., 2016). Bioimpedance analysis has been suggested as a non-invasive method to screen for diabetes mellitus (Jun et al., 2018).

Some studies have investigated the correlation between changes in glucose levels and bioimpedance (Das et al., 2017; Zhu F. et al., 2020; Jose et al., 2019; Satish et al., 2017). A recent study showed an inverse relationship between glucose concentration and the bioimpedance difference in blood volume (Li et al., 2018). Another study identified that a frequency band of below 40 kHz provided stable and reliable estimation for blood glucose based on bioimpedance (Takamatsu et al., 2021). Another study demonstrated a wearable prototype system for non-invasive glucose monitoring based on bioimpedance measurement and showed that certain parameters of bioimpedance were sensitive to changes in blood glucose levels (Liu et al., 2016). Another study proposed a hybrid technique that combined bioimpedance with near-infrared measurements to monitor glucose (Nanayakkara et al., 2018). Using machine learning (i.e., regression), the combination of the two measurements achieved better results based on Clarke’s error grid (i.e., 90% points in region A) when compared to the reference blood glucose. However, the study was limited to one participant.

In relation to blood trends, a recent study assessed non-invasive sensors that included bioimpedance measurements in detecting hypoglycemia among 20 patients with type 1 diabetes that underwent clamp procedures (Tronstad et al., 2019). The study revealed that bioimpedance plays a correcting role in the prediction when paired with other sensors. Bioimpedance of the skin has also been used to detect nocturnal hypoglycemic events (Lesko et al., 2018). Another study investigated the galvanic skin response (GSR) and correlated it with blood glucose levels (Snekhalatha et al., 2018), where a device was developed to acquire the GSR resistance and voltage data from 100 participants (i.e., 50 with diabetes). A negative correlation with GSR voltage and resistance was observed among diabetic patients. A recent study in 14 patients with type 2 diabetes collected bioimpedance data using a wearable ring over 2 weeks and used a gradient boosted model to estimate blood glucose levels and trends and showed excellent prediction performance with 99% of the values in zones A and B of the Parkes error grid (Sanai et al., 2021).

### 2.5 Sweat

Sweating is a normal physiological mechanism to regulate body temperature through evaporation, but it is also a key autonomic feature of hypoglycemia (Escolar et al., 2016). Sweat consists mostly of water but also contains sodium, chloride, potassium, lactate, and urea (Baker, 2019). Chemical components in the sweat have been utilized as biomarkers of disease, e.g., sweat chloride in cystic fibrosis (Accurso et al., 2014). A study used different non-invasive sensors (sweat, temperature, and ECG) in patients with type 1 diabetes and showed that measurement of sweating in combination with the ECG signal predicted the development of hypoglycemia (Elvebakk et al., 2018). Sweat also contain glucose at orders of magnitude lower in concentration (10–200 *μ*M) compared to blood glucose (Bariya et al., 2018), but require careful considerations to avoid contamination when collected from the skin surface (Moyer et al., 2012).

Wearable sensors can take advantage of the non-invasive nature of using sweat as a predictor of the human health status (Upasham and Prasad, 2020). There is a growing interest in developing sweat-based sensors and systems that are aimed to monitor health to help in the management of patients with diabetes (Hojaiji et al., 2020; Lee et al., 2017; Karpova et al., 2019). One study developed a microfluidic device using a cotton thread and filter paper paired with a smartphone to sense sweat glucose (Xiao et al., 2019). The device showed a linear trend in the 50–250 *μ*M range with a detection limit of 35 *μ*M and the nature of the sensor construction enabled it to be flexible, easy to integrate, and to be produced at relatively low-cost. Another study developed a biosensor based on a graphene oxide nanostructured composite deposited with gold and platinum nano-particles to detect glucose in human sweat (Xuan et al., 2018). When tested with sweat samples, the device showed a short response time and high linearity.

Katseli et al. (2021) demonstrated that a 3D printed electrochemical sweat sensor shaped like a ring was capable of monitoring sweat glucose in the range of 12.5–400 *μ*mol/L, which could be read by a smartphone. However, prototype testing was limited to one healthy volunteer. Similarly, Sempionatto et al. (2021) developed a touch-based sweat glucose sensor to estimate blood glucose. The electrochemical sensor consists of a sweat collecting layer, glucose biosensor, and a substrate that requires no sweat stimulation. The sensor achieved a high correlation (i.e., 0.95) with blood glucose and with all the points in the A and B regions on the Clarke error grid. Another study developed a tandem catalytic system for sweat glucose detection based on chemiluminescence with a high sensitivity and detection limit of 0.1 *μ*M when compared to solutions containing different glucose concentrations (Gao et al., 2021).

Smart wrist wearables are becoming an essential part of fitness and health monitoring. The convenience of wearing watches on the wrist only widened the adoption of such wearable devices (Guler et al., 2016). Several wrist wearable sensors have been developed to detect sweat glucose (Hong et al., 2018; Xuan et al., 2018; Lu et al., 2019). One study demonstrated a nonenzymatic wearable sensor that allowed the analysis of sweat glucose (Zhu X. et al., 2018). The sensor was made from a treated silver electrode coated with fluorocarbon-based materials. An integrated wristband containing the sensor provided continuous monitoring of sweat glucose and showed the results on a smartphone App. Their solution demonstrated the possibility of detecting glucose in the range of 30–1,100 *μ*M. However, the developed wearable was tested with samples acquired from participants only and no correlations with blood glucose were made. Another research group developed a fully integrated device to provide continuous monitoring of sweat glucose (Zhao et al., 2019). The device consists of flexible rechargeable batteries and photovoltaic cells that are used to power up the device (i.e., signal processing and display) using solar energy. Monitoring of sweat glucose is based on an electrochemical sensor connected to a controlling module. A small display is used to provide real time monitoring. The wearable displayed potential in detecting sweat glucose changes in the range of 50–200 *μ*M during different activities (e.g., running vs. biking).

### 2.6 Tears

The human eyes produce tears as a response to irritants, due to intense emotions, and to keep the ocular surface lubricated and protected. Tears are made of water, protein, lipids, and electrolytes (Dartt et al., 2005), but also contain traces of glucose that correlate with blood glucose levels (Chen et al., 1996; Zhang et al., 2011). The concentration of tear glucose is influenced by the method of collection. For example, a study found that onion-induced tear glucose concentration is up to 8 fold higher compared to one without stimulation (Taormina et al., 2007). This was attributed to the level of irritation in the onion-induced method that influenced the collected samples. Hence, a consistent method to collect the tears must be selected and careful consideration must be paid to the surrounding conditions (Rentka et al., 2017). Despite the complicated nature of collecting tear samples, there is a growing interest in the development of sensors capable of tear glucose monitoring (Strakosas et al., 2019; Chen et al., 2018).

A ratiometric fluorescent membrane capable of sensing tear glucose in the range of 0.1–10 mM has been developed (Duong et al., 2020). However, the testing conditions were limited to glucose solutions. Another study developed a low-cost, flexible, customizable, and disposable sensor strip based on engraved graphene to detect glucose in tears and saliva (Tehrani and Bavarian, 2016). The sensor displayed a promising sensitivity and low detection limit (i.e., 250 nM) when tested *in vitro*. However, no testing with real samples of human tears or saliva has been undertaken. A low-cost and non-enzymatic glucose sensor based on an inkjet printed electrochemical sensor was developed in another study (Romeo et al., 2018). The sensor was flexible and versatile in terms of fabrication, and demonstrated its ability in detecting glucose concentrations in human tears. However, tears were induced using onion and collected in glass capillaries. To overcome some of the limitations in tear glucose sensors, Belle et al. (2016) developed an integrated device that has a broad dynamic range, rapid analysis, low detection limit, works with small samples, and does not cause stress on the eye. The sensor could detect tear glucose in the range of 0.72 mg/dl to 111.6 mg/dl corresponding to 20 mg/dl to 600 mg/dl of blood glucose. However, the developed device was only tested in samples from an animal.

The development of wearable contact lenses capable of monitoring different physiological signs has gained considerable interest in recent years (Elsherif et al., 2018; Park et al., 2018; Kim et al., 2017). A contact lens comprised of three layers (silk fibroin, silver nanowires, and protected film) capable of sensing tear glucose in the range of 500 nM to 1 mM with a detection limit of 211 nM was developed by Lee W.-C. et al. (2021). Another contact lens was developed by Kim et al. (2017) which monitors tear glucose based on glucose oxidase linked to a graphene channel that can be read wirelessly by a coil. The *in vivo* experiments showed that the wearable sensor can detect tear glucose concentration when placed in a rabbit’s eye. Another study developed a fully integrated soft contact lens that contains glucose sensors, wireless circuits, and a display (Park et al., 2018). The developed smart contact lens is supposed to overcome some of the limitations of existing contact lens such as being brittle, blocking the vision, and requiring extra equipment to read the lens. The wearable was able to detect tear glucose when tested *in vivo* on a live rabbit.

An optical sensor embedded in a wearable contact lens was developed to provide continuous glucose monitoring (Elsherif et al., 2018). The reading of the sensor was based on smartphone camera readouts that correlated the reflected power of the diffraction with glucose concentration. The developed sensor could detect glucose concentration less than 50 mM with a 12 nm mM^−1^ sensitivity. However, no *in vivo* experiments were reported. They subsequently developed a bifocal contact lens containing a hydrogel glucose sensor that could detect tear glucose within the 0–3.3 mM range in artificial tears (Elsherif et al., 2021). Another study developed and clinically tested a flexible tear glucose biosensor (Kownacka et al., 2018). The coil-shaped sensor is 1.3 mm in diameter and 15 mm long and consists of a flexible coil made of electrodes arranged in parallel and an antenna wire that can transfer the readings to an external device through telemetry. The sensor was designed to be placed under the lower eyelid and was reported to not cause any irritation or abnormalities in a sheep’s eye. Clinical testing was conducted with six subjects who wore the sensors and CGMs (i.e., Abbott FreeStyle Libre) for 5 hours in a fasted state. On Clarke’s error grid, 95% of the data points for the sensor were in the A and B regions with 70% in region A, which was comparable to the performance of the CGM. Geelhoed-Duijvestijn et al. (2021) evaluated the same biosensor in 24 patients with type 1 diabetes by simultaneously measuring blood and interstitial glucose levels every 15 min compared to continuous tear glucose and a neural network based regression model was used to convert tear glucose to blood glucose. The performance of the device was comparable to the CGM with a mean absolute relative difference in glucose of 16.7 mg/dl.

### 2.7 Saliva

Saliva is a clear and slightly acidic secretion originating from the sublingual, submaxillary, parotid, and minor mucous glands and serves to lubricate and clean the oral tissues and assist in taste and digestion (Humphrey and Williamson, 2001; Dawes, 1974; de Almeida et al., 2008). Different concentrations of various electrolytes and minerals can be found in saliva including carbon dioxide, sodium, chloride, and potassium as well as traces of glucose (Schneyer et al., 1972; Thaysen et al., 1954; de Almeida et al., 2008). The excretion and concentration of salivary glucose has been found to be higher in diabetic patients compared to control subjects and there is a significant correlation between the concentration of glucose in the saliva and blood in patients with diabetes (Jurysta et al., 2009; Amer et al., 2001).

Interest in the use of saliva as a diagnostic fluid has grown considerably and several sensors have been developed (Nunes et al., 2015; Bihar et al., 2018). A study fabricated a non-enzymatic electrochemical sensor to measure salivary glucose with a working range varying from 0.5 to 50 *μ*g/ml and a detection limit of 1.9 *μ*g/ml and showed a highly significant correlation (r = 0.96) with blood glucose measured using the finger prick method (Diouf et al., 2019). Another study fabricated a saliva glucose optical sensor and showed that the glucose concentration increased the absorbance of light when tested at a wavelength of 630 nm (Jung et al., 2017). There was a good correlation between the glucose in blood and saliva. A disposable saliva glucose sensor based on dehydrogenase flavine-adenine dinucleotide was tested in nine healthy individuals and showed a detection range of 2.38–3.40 mg/dl corresponding to a blood glucose range between 90 and 143 mg/dl at a detection limit of 0.11 mg/dl (Lin et al., 2017). A salivary glucose biosensor utilized a new hydrogel film to improve glucose detection sensitivity by 130% with good linearity for glucose concentration between 0 and 50 mg/L (Zhang et al., 2021). Another study showed that enzymatic biosensors provided a linear relationship between electrical impedance and glucose concentration with the lowest detection limit being 14 *μ*M (Mercante et al., 2021). Another study developed bioconjugated nanoflowers which quickly (i.e., within 10 min) estimated salivary glucose concentration between 0.2–300 mg/dl (Shende and Kasture, 2021).

There have been attempts to incorporate saliva glucose sensors into devices used daily to measure glucose levels. One study embedded a saliva glucose sensor into a smart toothbrush which integrated a bronze based sensor to provide non-enzymatic electrochemical measurement of salivary glucose (Chen et al., 2019). The sensor showed a linear range from 0 to 320 *μ*M with a detection limit of 6.6 *μ*M. To test the sensor, saliva samples were acquired from five participants before and after meals. The sensor readings for the saliva glucose reflected well with the changes in blood glucose values. Embedding a saliva glucose sensor with wireless communication capabilities into a mouthguard has also been considered (Arakawa et al., 2016). The same team embedded a biosensor based on cellulose acetate into a mouthguard and was able to detect glucose concentration wirelessly in the range of 1.75–10,000 *μ*mol/L (Arakawa et al., 2020). However, the tests were limited to artificial saliva.

### 2.8 Acceleration

Involuntary shaking part of the human body, such as the hand, is one manifestation of the symptoms that are associated with hypoglycemia (Wild et al., 2007). The tremor that occurs during hypoglycemia is categorized under enhanced physiologic tremor and results from different mechanical and neuromuscular interactions (Rana and Chou, 2015). The enhanced physiologic tremor is usually more visible compared to normal tremors with a frequency that was estimated to be in the range of 5–14 Hz (Puschmann and Wszolek, 2011; Rana and Chou, 2015). Smart wearables can detect tremors and identify different patterns with the help of machine learning techniques, but have not been widely assessed to predict hypoglycemia (San-Segundo et al., 2020; Lonini et al., 2018; Zahed et al., 2018). A low-cost wearable device based on an accelerometer mounted on the index finger showed that it can detect tremor in the range of 10–14 Hz, but was not validated in patients with diabetes in relation to hypoglycemia (Abbas et al., 2018). In a study of seven patients with type 1 and type 2 diabetes, tremors in the frequency range of 10–14 Hz were easily distinguishable under fatigue, but no evaluation was undertaken in relation to hypoglycemia (Aljihmani et al., 2019).

Acceleration can be used to provide information about human activities and wearables with an accelerometer have found increasing use in health care applications such as detecting falls among the elderly (Janidarmian et al., 2017; Bagala et al., 2012). Given that severe hypoglycemia can markedly impair movement, information on physical activity may improve the prediction of glucose levels (Jaggers et al., 2019; Stenerson et al., 2014). Accelerometers have been used to plan physical activity levels in adults with type 1 diabetes (Keshawarz et al., 2018). In a study of ten adolescent athletes with type 1 diabetes, vigorous high intensity physical activity correlated with an increased risk of prolonged nocturnal hypoglycemia (Jaggers et al., 2019).

## 3 Machine Learning Techniques

A typical machine learning algorithm uses data to build a predictive model that can map a set of inputs to a desired output. The core element in machine learning is collecting enough data to be used in the training and evaluation of the predictive model. Once enough data is collected, a portion of this data is typically selected to be used to train and optimize a model’s parameters, and the remaining part is then used to evaluate the performance of the learned model. In glucose monitoring applications, wearable devices can be used to acquire physiological data as inputs while a CGM device is used to acquire the output or target values. Machine learning techniques have been considered for several glucose monitoring applications such as predicting current glucose levels, forecasting future values, and classifying ongoing trends.

**FIGURE 4:**
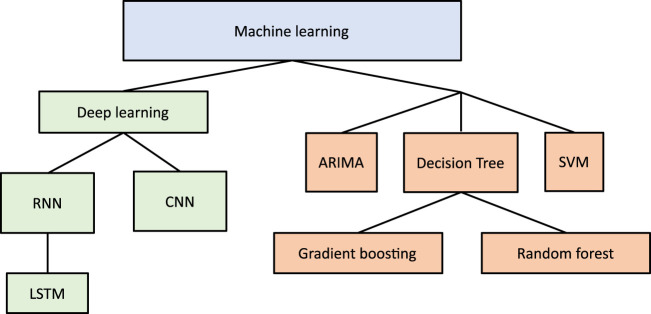
Machine learning algorithms utilized in glucose monitoring applications over the past five years.

This section presents the different machine learning techniques that have been used for blood glucose monitoring and trends detection (Figure 4). A summary of the surveyed studies is provided in Table 2.

**TABLE 2:** A summary of machine learning based blood glucose monitoring contributions.

Study	Inputs	Data Used	Algorithm	Key Results
G et al. (2018)	ECG signals used in the analysis of HRV	Data acquired from 20 healthy participants and 20 with diabetes in supine position	CNN and a hybrid network of CNN-LSTM	CNN-LSTM achieved 95.1% in distinguishing diabetes
Idrissi and Idri (2020)	Blood glucose levels using a CGM	Dataset from 10 T1DM patients	CNN and LSTM	CNN outperformed LSTM in blood glucose prediction tests
Zhu et al. (2018a)	Dataset containing glucose levels, insulin dosages, and carb intake	Data of 6 participants with T1DM collected over 8 weeks	CNN	The best CNN model achieved an RMSE of 21.72 mg/dl in predicting glucose levels
Porumb et al. (2020a)	ECG signals	Data acquired from 8 healthy elderly participants	CNN with autoenecoders	Achieved 90% in nocturnal hypoglycemia detection
Shahid et al. (2021)	CGM, carbohydrates, and insulin	Simulated data representing 10 patients with T1DM	CNN with gated recurrent unit neural networks	RMSE of 6.04 mg/dl for the 30 min prediction horizon
Samir et al. (2021)	CGM, food, and human activity	Data acquired from patients with diabetes	CNN	Classifying hyperglycemia with 93.2% accuracy
Zhu et al. (2020b)	Meal intake, glucose levels, and insulin dosage	Used simulated and real datasets of T1DM subjects	Dilated RNN and transfer learning	Forecasting future glucose levels with an RMSE of 18.9 mg/dl
Faruqui et al. (2019)	Data contained glucose levels, diet, and physical activity	Data acquired from 10 subjects with T2DM monitored for 6 months	LSTM based structure	84.12% of next day predictions in zone A of Clark’s error grid
Gu et al. (2017)	CGM, Insulin, food, and physical activity	Dataset collected from 112 participants (35 healthy, 38 with T1DM, and 39 with T2DM)	RNN	Achieved a blood glucose inference accuracy of 82.14%
Nasser et al. (2021)	CGM	Data acquired from 10 patients with T1DM	RNN with restricted boltzmann machines	RMSE value of 15.59 mg/dl for 30 min prediction horizon
Eom and Lee (2022)	CGM, insulin, and carbohydrate	Dataset of 30 patients	Weibull Time To Event RNN	Predicting future episodes of hypoglycemia with an RMSE of 12.56 mg/dl
Zhang et al. (2017)	Physiological parameters and metabolic rate	Data collected from healthy subjects, senior citizens, and patients with diabetes	DT and ANN	Blood glucose prediction with 88.53% accuracy
Tsai et al. (2020)	PPG signals	9 subjects with T2DM	DT based structure (Random forest)	90% accuracy in predicting glucose
Maritsch et al. (2020)	HRV using PPG smartwatch	One participant with T1DM	DT based structure (Gradient boosting)	82.7% accuracy in detecting hypoglycemia
Reddy et al. (2019)	HR, physical activity, and glucose levels	43 subjects with T1DM during aerobic exercise	DT	86.7% accuracy in predicting hypoglycemia during exercises
Aashima et al. (2022)	CGM	Simulated CGM data from 40 participants with T1DM	DT and Adaboost	RMSE value of 2.204 mg/dl in predicting blood glucose levels
Sankhala et al. (2022)	Sweat glucose	Three participants	DT	RMSE value of 0.1 mg/dl in estimating sweat glucose
Habibullah et al. (2019)	NIR signals	Used artificial blood samples with different glucose concentration	SVM	77.5% accuracy with PCA
Malik et al. (2016)	Salivary electrochemical properties	175 participants including participants with T2DM	SVM, ANN, and logistic regression	SVM achieved 85% accuracy in detecting fasting blood glucose
Marling et al. (2016)	Heart rate, galvanic skin response, CGM, and temperature	One participant with T1DM	SVM	SVM with a linear kernel showed a promising performance
Bertachi et al. (2020)	CGM, HR, steps, sleep, and calories	10 subjects with T1DM	SVM and MLP	SVM achieved the best results in predicting nocturnal hypoglycemia
Shokrekhodaei et al. (2021)	Intensity data of an optical sensor	In aqueous solutions containing glucose	SVM	RMSE value of 12.5 mg/dl and 99.55% accuracy
Yang et al. (2019)	CGM	100 participants with T1DM and T2DM	ARIMA	9.4% false rate in predicting future blood glucose trends
Prendin et al. (2021)	CGM	Partial data from 141 patients with T1DM	ARIMA	RMSE of 22.15 mg/dl in 30 min prediction horizon and also in hypoglycemia detection with 64% precision and 82% recall

### 3.1 Artificial Neural Network

An ANN consists of interconnected layers of perceptrons that can learn patterns of data by adjusting numerical weights attached to each connection. Several popular ANN architectures have been considered for the purpose of blood glucose monitoring (Ali et al., 2018; Mhaskar et al., 2017).

#### 3.1.1 Convolutional Neural Network

**FIGURE 5:**
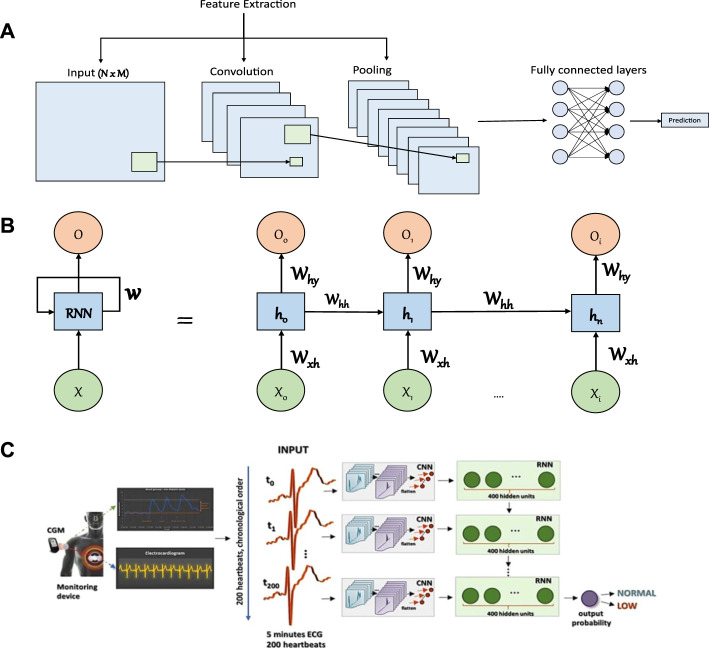
Illustrations of commonly considered artificial neural networks in glucose monitoring applications and an example of a study that considered a combined architecture. **(A)** Illustration of a CNN model. The first layer is the input layer which holds values of the input data that is followed by a convolution layer, which serves to extract meaningful patterns in partial regions of the input. Next, comes the pooling layer that will perform a downsampling operation that reduces the number of parameters. The extracted features are then passed to a fully connected layer to make the final prediction. **(B)** Illustration of RNN model. The RNN model consists of an input layer (*X*), hidden layers to model sequential information (from *h*_0_ to *h*_*n*_ where *n* is the number of hidden layers), and an output layer (*O*). The structure is connected with weights that link the input layer to the first hidden layer (*W*_*xh*_), the hidden layers together (*W*_*hh*_), and the last hidden layer to the output layer (*W*_*hy*_). **(C)** An example of a study that considered the application of CNN and RNN to predict the occurrence of hypoglycemia based on ECG data (Adapted with permission from Porumb et al. (2020b)). The isolated heartbeats were combined into segments of 5 min each and each segment has been assigned a label (i.e., low or normal glucose level) based on the recorded CGM value.

CNN is a type of ANN mainly used for processing and recognition of grid like data (e.g., images) and typically consists of three types of building blocks (LeCun et al., 2015). The first two building blocks (i.e., convolution and pooling layers) perform feature extraction while the third (i.e., fully connected layer) matches the extracted features with outputs or classes. The stacking of these blocks constitutes the CNN architecture (Figure 5A). To make a prediction, the first layer in CNN receives a vector of input values that can either be spatially related such as images, or short sequences of time series data such as multi-dimensional biometric data. These values are then passed to several layers that perform two operations; convolutions, and downsampling. In the convolution layers, specific features are extracted from nearby inputs by matching learned meaningful patterns with the sequence of data that are fed into the layer. The results of this operation are then forwarded to a next layer which chooses the patterns that were most apparent (i.e., max-pooling). This operation is repeatedly done depending on the number of layers used. Finally, the resulting patterns are fed into a fully connected ANN that produces a prediction based on the information presented on the last preceding layer. The number of patterns to match in each layer (i.e., filters) and number of stacked layers are hyper-parameters that are tuned based on the desired prediction performance and computational cost.

CNN has been utilized in many applications, e.g., heart-beat classification (Acharya et al., 2017), COVID-19 detection (Wang et al., 2020), and glaucoma detection (Chen et al., 2015). Models based on CNN have also been developed to predict and forecast blood glucose levels and trends (Swapna et al., 2018; Idrissi and Idri, 2020; Kamalraj et al., 2021). A personalized CNN model employing a fine-tuning strategy improved the prediction horizon performance compared to standard CNN when evaluated using CGM data (Seo et al., 2021). Among six patients with type 1 diabetes, a dataset containing insulin dose, carbohydrate intake, and glucose levels for 8 weeks was used to train and benchmark a blood glucose forecasting model based on casual dilated CNN (Zhu T. et al., 2018). Preprocessing (interpolation, extrapolation, and filtering) was performed to compensate for missing values and to clean the data (e.g., remove noise). The inputs to the neural network were the recorded CGM, insulin, carbohydrate intake, and time index mapped to 256 classes that represent a change of 1 mg/dl between each class. The results showed promise with an average root mean squared error (RMSE) of 21.72 mg/dl for a 30 min prediction horizon. A hybrid model consisting of CNN and gated recurrent unit neural networks has been proposed to reduce the error rate in predicting blood glucose levels (Shahid et al., 2021). Based on simulated data, the proposed model achieved an RMSE of 6.04 mg/dl for the 30 min prediction horizon and 8.12 mg/dl over 60 min.

A more recent study considered a combination of CNN with autoencoders to detect nocturnal hypoglycemia (Porumb et al., 2020a). The study used a non-invasive wearable (i.e., ECG) and CGM to acquire data from 25 elderly subjects under controlled conditions for up to 36 h. The collected data were used to train and evaluate personalized deep learning models. The best trained model was able to distinguish low blood glucose trends with an accuracy of 90% in the test dataset. Samir et al. (2021) proposed another CNN based model to predict blood glucose trends by classifying food and human activity and classified hyperglycemia with an accuracy of 93.2% based on the D1NAMO dataset of diabetic patients using CGMs alongside food images and acceleration data (Dubosson et al., 2018). Another study also considered ECG wearable to classify blood glucose into three levels, namely, low, moderate, and high (Li et al., 2021). The study used a finger pricking device (i.e., Accu-Chek) to acquire the blood glucose data from 21 healthy and prediabetic adult participants during fasting and oral glucose tests. A CNN-based model was able to classify low glucose (87.94%), moderate glucose (69.36%), and high glucose (86.39%) in the testing datasets.

#### 3.1.2 Recurrent Neural Network

RNN is a class of ANNs with feedback signals developed to learn sequential ordered data (i.e., time series) or time-varying patterns such as that found in speech (Medsker and Jain, 1999). The prediction in RNN relies on previous information maintained internally. The hidden layers act like a memory that captures the information about a sequence. RNN models consist of interconnected layers of neurons just as in normal ANNs (Figure 5B). The difference in RNN is their ability to take into account information from previous predictions. Specifically, each hidden layer also considers the outputs of hidden layers from preceding predictions. This allows the network to capture information from ordered sequences of data. The learning in a standard RNN structure might be limited and hindered due to the vanishing gradients problem, hence, a structure based on RNN (e.g., long-short term memory (LSTM)) is usually used to combat that (Hochreiter and Schmidhuber, 1997).

Several applications used RNN based models to recognize different sequences in human activities (Pienaar and Malekian, 2019), emotion recognition based on videos (Fan et al., 2016), and the characterization of unwanted interactions with small robots (Alhaddad et al., 2020). RNN has also been considered for blood glucose prediction among patients with diabetes (Sun et al., 2018; Mirshekarian et al., 2017; Martinsson et al., 2018; Aliberti et al., 2019). A recent study developed a model based on a RNN structure with the help of transfer learning to forecast future glucose levels (Zhu T. et al., 2020). They believed that forecasting of future blood glucose will help to enhance the CGM and insulin pump systems by calculating the optimum insulin doses avoiding any adverse events. The study considered simulated and actual datasets containing information on meal intake, CGM readings, and insulin dosage to evaluate the developed model. The results showed a good 30 min forecasting performance of the developed model (RMSE = 18.9 mg/dl) compared to other tested algorithms.

In another work, an LSTM-based algorithm was applied to 6-months data on diet, glucose levels, and physical activity in 10 patients with type 2 diabetes to forecast daily glucose concentrations (Faruqui et al., 2019). They were able to predict the next day glucose concentrations with 84.12% of the predicted values in zone A based on Clark error grid when compared to the true values of blood glucose. However, the study was limited to a small sample size and was affected by individual variations and data collection challenges. An inference system based on a smartphone to monitor blood glucose non-invasively was developed by Gu et al. (2017). They collected data about insulin, drug dosage, food intake, sleep quality, and physical activities along with CGM. The authors evaluated their system on 112 subjects and the implementation of RNN achieved an accuracy of 82.14% in tracking blood glucose levels into four classes. A recent study also assessed the ability to estimate future (i.e., 30 min prediction horizon) blood glucose levels using an IoT device with CGM sensor, an application layer protocol, and prediction model on the cloud (Nasser et al., 2021). Based on a model consisting of RNNs and restricted boltzmann machines, the proposed system achieved an RMSE value of 15.59 mg/dl in data acquired from ten patients with type 1 diabetes. Another recent study proposed using Weibull Time To Event RNN (i.e., WTTE-RNN (Martinsson, 2017)) to predict future episodes of hypoglycemia and reported an RMSE of 12.56 mg/dl (Eom and Lee, 2022).

### 3.2 Decision Tree

A decision tree (DT) is a divide-and-conquer method that partitions data such that it becomes easy to classify. A typical DT model consists of nodes that split the data based on attribute-value combinations. Data are split repeatedly until a given criteria is satisfied (e.g., similarity of data in each partition). DT has been applied to extract and analyse information from large datasets (i.e., data mining) and in machine learning (e.g., classification and regression) (Hastie et al., 2009; Navada et al., 2011). Classification trees are used when the target values are discrete while regression trees are used when the target values are continuous. Compared to other machine learning techniques, DT has the advantage of model interpretability. It provides an insight on the most influential data attributes related to the task at hand and helps to plan future experiments (Myles et al., 2004). Different forms of DT (e.g., classification, regression, and forests) can be considered depending on the problem and the desired output (Navada et al., 2011).

Decision trees were used as prediction models for risk factor interactions in diabetes and to identify subjects with impaired glucose metabolism (Ramezankhani et al., 2016; Hische et al., 2010). Different DT models have been used to identify vital indicators in relation to blood glucose prediction (Liu et al., 2020). One study developed a non-invasive system to detect blood glucose levels based on the conservation-of-energy method and physiological parameters (Zhang et al., 2017). The study acquired data samples from 400 participants (i.e., healthy and diabetic patients) that were then used in the model development and evaluation. Using an algorithm that combines a DT and neural network, the proposed approach was able to provide a blood glucose prediction with an accuracy of 88.53% in classifying new blood glucose samples.

A recent study considered a wearable device to estimate blood glucose based on photoplethysmography (PPG) signals (Tsai et al., 2020). The data were acquired from 9 patients with type 2 diabetes in a stable physical position (i.e., sitting). The blood glucose levels were acquired using a finger prick device (i.e., Accu-Chek). A machine learning algorithm based on decision tree (i.e., random forest) was considered to build personalized and generalized models that achieved 80% and 90%, respectively when tested with unseen data. Another study recently evaluated several ensemble machine learning models to provide a generalized blood glucose prediction (Aashima et al., 2022). Simulated CGM data from 40 participants with type 1 diabetes were used to train and test the models. A combined model (i.e., DT and Adaboost) outperformed the other models and achieved an RMSE value of 2.204 mg/dl when evaluated against test samples. Another study developed a non-invasive platform to measure sweat glucose periodically and utilized a machine learning algorithm to generate sweat glucose readings from the discrete values (Sankhala et al., 2022). A DT model was considered to provide sweat glucose readings based on the raw impedance signal, relative humidity, and temperature and the regression model achieved an RMSE value of 0.1 mg/dl in three participants.

DT based models were also utilized to predict blood glucose trends, such as hypoglycemia. One study proposed using a machine learning model based on decision trees (i.e., gradient boosting) to detect hypoglycemia using a wearable device (Maritsch et al., 2020). Using features acquired from the heart variability rate, the study developed a machine learning model based on the data acquired from one participant with type 1 diabetes. The results of the unseen samples demonstrated the possibility of detecting hypoglycemic events with 82.7% accuracy. Reddy et al. (2019) investigated the possibility of predicting hypoglycemia in 43 adults with type 1 diabetes who were performing aerobic exercise. The extracted features included physical activity, heart rate, anthropometric data, energy expenditure estimate, glucose readings, and physical activity. The results of two developed models based on decision trees showed promising results in predicting hypoglycemia with an accuracy of 86.7%.

### 3.3 Support Vector Machine

SVM is a supervised machine learning technique used in classification and regression. In classification problems, the SVM learns from the labeled training data how to best categorize data that belongs to one of two classes by finding the optimal hyperplane that separates them (Hearst et al., 1998; Brereton et al., 2010). The separation in SVM can be based on a linear, or non-linear combination of features depending on the complexity of the task at hand and feature dependencies. In case of a non-linear SVM, kernel functions are used to transform a problem to a linearly separable one by projecting the problem from a low-dimensional space to a high-dimensional one (Patle and Chouhan, 2013). SVM is also used in linear and non-linear regression. The principle of SVM for regression is to find a flat function that satisfies a deviation criterion from the target outputs with less restriction to minimize the errors (Smola and Schölkopf, 2004). In case of a non-linear regression, a similar technique to that used in classification is applied.

SVM has been considered in different areas and in many different applications such as in cancer genomics (Huang et al., 2018), chemistry (Ivanciuc, 2007), autism therapy (Alban et al., 2021), and bioinformatics (Byvatov and Schneider, 2003). A custom-built optical sensor was used to investigate the relationship between wavelengths and glucose concentrations in aqueous solutions containing different glucose concentrations ranging from 40 to 250 mg/dl (Shokrekhodaei et al., 2021). Intensity data based on the four optimal wavelengths (i.e., 485, 645, 860 and 940 nm) were considered in training a classifier and a regression model to predict either a discrete range (i.e., 21 classes) or a continuous value, respectively. A classifier based on SVM achieved the best results with an RMSE value of 12.5 mg/dl and 99.55% of the predictions in zones A and B on the Clarke error grid.

SVM has also been applied in glucose monitoring and long-term diabetes outcome prediction (Barman et al., 2010; Li and Fernando, 2016; Abbas et al., 2019). A study surveying machine learning techniques for blood glucose prediction found that a regression model based on SVM performed best in the short term forecasting of blood glucose (Mayo et al., 2019). Based on near infrared (NIR) spectroscopy data in ten discrete artificial blood samples, SVM alone achieved an accuracy of 67.5% when evaluated with the testing dataset, but when paired with principle component analysis (PCA) it improved to 77.5% (Habibullah et al., 2019). Another study tested several machine learning techniques that included SVM in detecting fasting blood glucose based on measuring the electrochemical properties of saliva (Malik et al., 2016). The fasting blood glucose was measured on venous plasma using an automatic biochemical analyzer and used as the target or true value. The electrochemical parameters of saliva (e.g., pH, conductivity, and sodium concentration) and fasting glucose were collected from 175 participants of whom half had diabetes. A SVM classifier trained with 70% of the data using the radial basis kernel function achieved the best results in distinguishing high vs. low fasting blood glucose levels in the remaining unseen testing data (i.e., accuracy and F1 score of 85%). Support vector regression has been used to predict future blood glucose levels using CGM in 12 patients and the best trained model achieved an RMSE of 12.95 mg/dl for a prediction horizon of 60 min (Hamdi et al., 2018).

A few studies have also considered using SVM to predict the occurrence of hypoglycemia among patients with type 1 diabetes. For example, one study used a non-invasive wearable device that measured air temperature, heart rate, and galvanic skin response to acquire data from one participant with type 1 diabetes for 2 months (Marling et al., 2016). The blood glucose data were acquired using a Dexcom CGM device and contained 34 hypoglycemic events, each lasting for 10 min or more. SVM with a linear kernel achieved the best performance, but the results were limited to one participant. Another study used a CGM device (FreeStyle Libre) in 10 participants with type 1 diabetes over 12 weeks and showed that SVM achieved a high sensitivity (78.75%) and specificity (82.15%) to predict nocturnal hypoglycemia (Bertachi et al., 2020).

### 3.4 Autoregressive Integrated Moving Average

ARIMA is a linear time series model used to predict or forecast future values based on past values. It is a function that includes differencing operators, and autoregressive and moving average terms (Box et al., 2011; Rodríguez-Rodríguez et al., 2019). ARIMA is considered as a generalized model of the autoregressive moving average (ARMA) as it incorporates a broad range of non-stationary series (Brockwell et al., 2002). ARIMA has been used to predict traffic noise pollution (Garg et al., 2015), web applications workload (Singh et al., 2019), and traffic flow (Kumar and Vanajakshi, 2015). Models based on ARIMA have also been considered in the prediction of blood glucose levels (Rodríguez-Rodríguez et al., 2019). A study used ARIMA to assist in predicting future blood glucose trend changes for hypoglycemia and hyperglycemia (Yang et al., 2019). Based on a combination of the ARIMA model and an adaptive algorthim, the study developed a prediction framework using continuous glucose monitoring (CGM) data from 100 patients with type 1 and type 2 diabetes. Their model provided early alarms with a 9.4% false rate at a sensitivity of 100%. Another recent study utilized CGM data to compare the 30 min prediction horizon performance of thirty linear and nonlinear algorithms (Prendin et al., 2021). Individualized ARIMA was the best linear algorithm in terms of accuracy with an RMSE of 22.15 mg/dl and also in hypoglycemia detection with 64% precision and 82% recall.

## 4 Discussion

The past five years has witnessed considerable advances in the development of sensors that measure different modalities which correlate with blood glucose. Glucose levels in tears, saliva, and sweat are related to the blood glucose levels and the advances in non-invasive wearable technology have not only allowed an estimation of blood glucose levels, but also the prediction of hypoglycemia. The developed wearables have targeted the heart, skin, eyes, and mouth using various technologies such as electromagnetic and bioimpedance. Some of these solutions have been augmented with machine learning techniques which have yielded promising outcomes, especially in hypoglycemia prediction. However, there remain considerable challenges before these devices can achieve FDA approval.

### 4.1 Participants and Testing Conditions

A major limitation in the studies to date is the number of participants with the majority of studies being limited to a few participants. This limitation clearly affects their clinical use and hinders the generalizability of the findings. Furthermore, some studies did not even undertake trials in patients with diabetes, which limits the applicability of the proposed solutions to the target end-users. Many of the studies reported only the initial or exploratory results of the developed sensors or wearable prototypes. The majority of studies were conducted under controlled settings and were limited to subjects of a younger age. Future studies should recruit larger numbers of participants with a wider age range and especially patients with type 1 or type 2 diabetes.

### 4.2 Bodily Fluids Glucose Sensors

The glucose levels in these bodily fluids were reported to correlate with blood glucose concentrations, but the glucose concentrations in these bodily fluids are low compared to that found in the blood. This represents a major challenge for the development of sensors and technologies that rely on bodily fluids to estimate blood glucose levels and trends. Hence, extra considerations such as the enhancement of sensitivity and interference elimination become crucial in the development of such sensors (Yao et al., 2011). Another challenge is the way a body fluid sample is collected (e.g., stimulated or unstimulated) as it was found to influence the content of the acquired sample. For example, diagnostic biomarkers were found to be higher in unstimulated saliva compared to stimulated saliva (Miller et al., 2010). A study has also shown that unstimulated saliva is highly accurate for predicting blood glucose (Cui et al., 2021). Hence, the collection techniques require standardization to avoid influencing the contents of the collected samples (Robson et al., 2010; Rentka et al., 2017). Contamination in the collected samples is another challenge that must be addressed as contaminated samples will generate false reading on tear glucose levels (Aihara et al., 2020).

### 4.3 Noise Susceptibility and Physiological Effects

Some of the physiological sensors were found to be influenced by daily circadian rhythms in heart rate and sweating (Elvebakk et al., 2018). Error can also arise due to non-linear dynamics of physiological signs being measured, making sensors more susceptible to error and high noise levels (Zanon et al., 2017; Gadaleta et al., 2019). Some studies have reported the adverse impact of environmental conditions (e.g., pH, humidity, and temperature) (Park et al., 2006; Elsherif et al., 2018). The time delay of a sensor or wearable device must be minimized to capture the rapid changes in blood glucose (Marcus et al., 2020). For patch-like sensors, the multidirectional stretchability of the sensor is an issue that needs careful considerations to ensure accuracy and stability under multi stretching cycles (Bae et al., 2019). Furthermore, reproducibility of sensor characteristics can be influenced by the fabrication processes (Mano et al., 2018). Finally, the majority of devices remain in the development stage and require extra-large devices to be connected to read out the signal and provide the filtration needed (Hanna et al., 2020). Electronics and mechanical miniaturization in wearable sensors represents a major limitation that requires further technological development and optimization (Elsherif et al., 2018).

### 4.4 Data Acquisition and Model Generalizability

A key challenge in machine learning is acquiring enough comprehensive data to train and test a model that can then be generalized to a wider population. Many studies were limited in terms of the number of participants, duration of the study, and the incidence of clinically relevant severe hypoglycemia. Additionally, most studies established their methods and techniques in relation to CGM data that may be limited in certain scenarios. There is a need to include a larger population of patients, especially with diabetes, to account for the inter-individual differences and to establish better validation of the proposed solutions. To capture meaningful data, the duration of trials for data acquisition and the incidence of hypoglycemia need to be sufficient to avoid unbalanced or skewed data (Marling et al., 2016). In the case of imbalanced data, oversampling techniques can address this and improve the accuracy (Mayo et al., 2019). Acquiring data from the same participant for longer periods allows the machine learning algorithm to combat intra-individual differences and increases overall prediction performance (Eljil et al., 2016). The reliance on data annotated by the participants is an issue in acquiring accurate information as it depends solely on their commitment (Bertachi et al., 2020). The experimental conditions should not be controlled to allow data acquisition of more realistic daily life settings. Future studies should also include a wider set of physiological parameters to investigate their individual or collective effect on estimating blood glucose trends using machine learning techniques. Additionally, data assimilation techniques could be considered in conjunction with machine learning techniques using data acquired from several sources and wearables (Albers et al., 2017; Albers et al., 2018).

### 4.5 Model Interpretability

Although machine learning algorithms have great predictive potential, the majority of these algorithms are black box models and lack the means to explain their predictions. The interpretability requirement of a machine learning model in health care is crucial (ElShawi et al., 2020). Clinicians need to make an informed decision based on a prediction but need to provide a proper explanation. Whilst wearable sensors are capable of estimating blood glucose trends based on physiological changes, such predictions without an underlying explanation may confuse the patient and render the wearable unreliable (Maritsch et al., 2020). Future studies must evaluate and incorporate interpretability techniques into their proposed solutions while making sure that the information acquired by the sensors are presented in a user-friendly interface (ElShawi et al., 2020). A support decision system along with the prediction was suggested to allow patients to provide feedback to evaluate the performance of a machine learning algorithm in real-life scenarios (Bertachi et al., 2020). Another consideration of an algorithm is the computational cost and energy. A wearable device has limited resources to perform complex operations and to operate for a long duration. Hence, feature engineering and data reduction techniques are needed to reduce the computational cost and improve energy efficiency (Gómez-Carmona et al., 2020; Al-Jarrah et al., 2015).

## 5 Conclusions and Future Directions

This review highlights the considerable progress made over the past five years in the area of non-invasive blood glucose monitoring using wearable technologies and sensors alongside machine learning algorithms. The devices have varied modalities and adopted technologies with novel approaches utilizing machine learning techniques to provide meaningful interpretations of multiple physiological parameters. However, there remain considerable limitations and challenges that hinder FDA approval and more widespread adoption of such technologies in patients. We therefore recommend future studies to focus on the following areas:

1. The recruitment of a larger number of participants especially patients with diabetes to validate the proposed techniques for use in the clinical arena.
2. Validating glucose sensors by adequate collection without contamination of bodily fluids.
3. Miniaturization of electronics and sensors for practical deployment.
4. Consistent evaluation of algorithms in personalized vs. generalized scheme, where a model is trained either on a target individual or a group of subjects.
5. The utilization of a wider set of physiological parameters for machine learning and data assimilation techniques while establishing effectiveness of each sensor’s contribution.
6. Investigating means to reduce computational cost and energy in wearable devices.
7. The development of interpretable machine learning models.
